# Isolation and analysis methods of extracellular vesicles (EVs)

**DOI:** 10.20517/evcna.2021.07

**Published:** 2021-03-30

**Authors:** Zheng Zhao, Harshani Wijerathne, Andrew K. Godwin, Steven A. Soper

**Affiliations:** ^1^Bioengineering Program, University of Kansas, Lawrence, KS 66045, USA.; ^2^Center of BioModular Multiscale Systems for Precision Medicine, Lawrence, KS 66045, USA.; ^3^Department of Mechanical Engineering, Temple University, Philadelphia, PA 19122, USA.; ^4^Department of Chemistry, University of Kansas, Lawrence, KS 66045, USA.; ^5^Department of Mechanical Engineering, University of Kansas, Lawrence, KS 66045, USA.; ^6^KU Cancer Center, University of Kansas Medical Center, Kansas City, KS 66160, USA.; ^7^Ulsan National Institute of Science & Technology, Ulju-gun, Ulsan, 44919, South Korea.

**Keywords:** Extracellular vesicles, molecular cargo, microfluidics, nanoparticle tracking analysis, resistive pulse sensing

## Abstract

Extracellular vesicles (EVs) have been recognized as an evolving biomarker within the liquid biopsy family. While carrying both host cell proteins and different types of RNAs, EVs are also present in sufficient quantities in biological samples to be tested using many molecular analysis platforms to interrogate their content. However, because EVs in biological samples are comprised of both disease and non-disease related EVs, enrichment is often required to remove potential interferences from the downstream molecular assay. Most benchtop isolation/enrichment methods require > milliliter levels of sample and can cause varying degrees of damage to the EVs. In addition, some of the common EV benchtop isolation methods do not sort the diseased from the non-diseased related EVs. Simultaneously, the detection of the overall concentration and size distribution of the EVs is highly dependent on techniques such as electron microscopy and Nanoparticle Tracking Analysis, which can include unexpected variations and biases as well as complexity in the analysis. This review discusses the importance of EVs as a biomarker secured from a liquid biopsy and covers some of the traditional and non-traditional, including microfluidics and resistive pulse sensing, technologies for EV isolation and detection, respectively.

## INTRODUCTION

Biomarkers secured from a liquid biopsy are generating significant interest in the research and medical communities due to the minimally invasive nature of acquiring them and the fact that they can enable precision medicine, which seeks to manage a variety of diseases using molecular signatures unique to the patient^[1,2]^. EVs are one of the many liquid biopsy markers that can be secured from a clinical sample, such as whole blood, saliva, urine, and cerebral spinal fluids.

**Figure 1:**
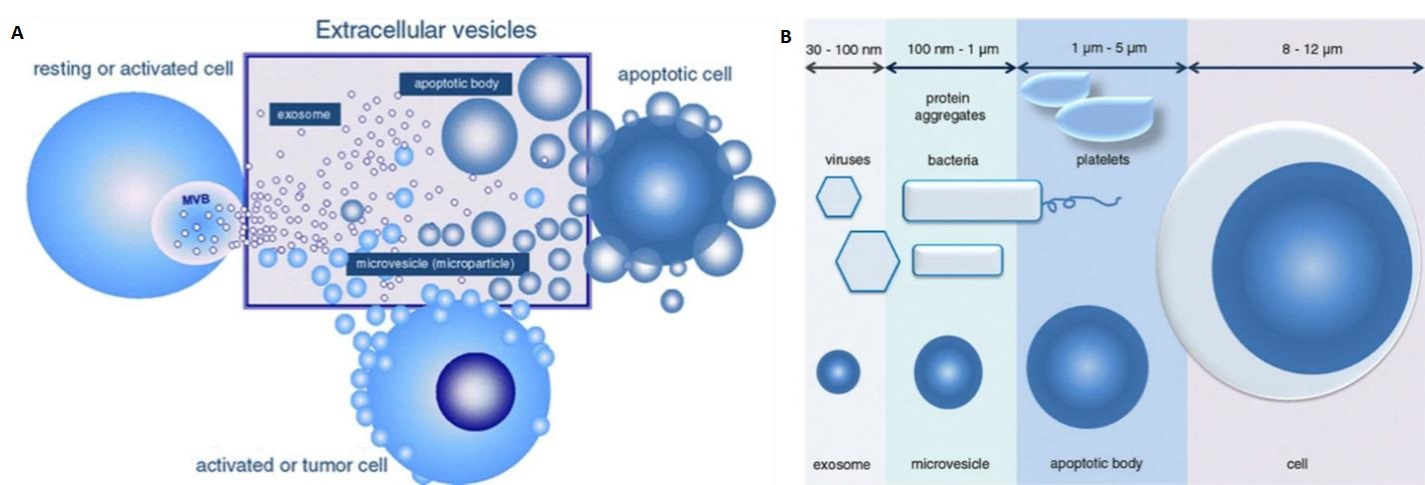
(A) Sub-types of extracellular vesicles including microvesicles, exosomes, and apoptotic bodies. (B) Size ranges of the three sub-types of extracellular vesicles of which exosomes are the smallest with a range from 30 to 150 nm. Microvesicles range from 100 to 1000 nm in size, but the size ranges from 100 to 400 nm when they are present in the circulatory system. Apoptotic bodies range from 1 µm up to 5 µm in size (Reproduced from^[10]^).

Biological cells release vesicles of varying sizes through both the endosomal pathway or budding/blebbing from the plasma membrane. These vesicles are known by different names, including microvesicles (MVs), exosomes, and apoptotic bodies,which are collectively called EVs^[3] ^[Figure 1A]. The particular subtype classification of EVs is based on their cellular origin and biogenesis^[4]^. MVs are heterogeneous, membrane-bound vesicles generated by budding/blebbing from the plasma membrane^[5]^, and range from 100 nm to 1 µm in size. On the other hand, exosomes are the smallest category in the EV family with sizes ranging from 30-150 nm and are released into the extracellular environment after the fusion of late endosomes/multivesicular bodies with the plasma membrane. Finally, apoptotic bodies are generated due to programmed cell death called apoptosis, and range from 1-5 µm in size. Figure 1B shows the size variations of the different types of EVs^[6]^. 

EVs contain variable components including lipids, carbohydrates, cytokines, proteins, and nucleic acids, in particular RNAs^[7]^. Both the surface and intra-vesicle material of EVs originate from their host cells making EVs suitable biomarkers for disease management, such as diagnosis, monitoring response to therapy, and determining disease recurrence^[6]^. However, before analyzing EVs they must typically be “enriched” from the clinical sample because they are typically a vast minority in a mixed population.

There is now a pressing need to “enumerate” EV biomarkers and analyze their molecular contents to provide relevant information for disease detection and management. The challenge with liquid biopsy markers is the mass limits they imposed on the molecular assay. Even though EVs are high in numbers (10^6^-10^13^ EVs per mL of plasma), their small size limits the molecular content within a single EV. For example, a 150 nm (diameter) EV may contain approximately 10,000 nucleotides of nucleic acids. In addition, components present in a sample may interfere with the molecular processing, and enrichment can obviate this issue.

Enrichment and detection techniques can take advantage of either the physical properties of the EVs (size, density, electrical properties, and morphology) or their biological properties (antigen expression). The next few sections will focus on reviewing EVs’ physical properties, intra-vesicle contents, diagnostic and therapeutic applications, isolation methods, and direct detection methods.

## TYPES OF EVS

### Microvesicles

Microvesicles are heterogeneous, membrane-bound vesicles that are 100 nm to 1 µm in size and are released from the surface of many cell types, including embryonic stem cells, neurons, and astrocytes, under both physiological and disease conditions^[8]^. MV biogenesis takes place through direct outward blebbing and pinching of the plasma membrane^[8]^. Platelets, red blood cells, and endothelial cells have been verified as a significant source of MV secretion, and tumor cells also constantly release MVs^[9,10]^. MVs are important in altering the extracellular environment, intracellular signaling, and facilitating cell invasion through cell-independent matrix proteolysis^[11]^. MVs can also contribute to the pro-invasive character of tumors and increase oncogenic intercellular transformation^[12,13]^. Differential centrifugation and flow cytometry are the commonly used isolation and detection methods, respectively^[10,14]^.

### Exosomes

**Figure 2:**
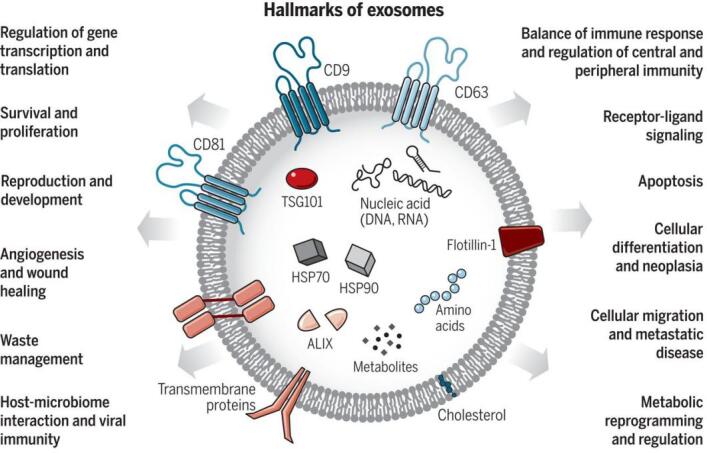
Exosomes are small EVs (sEVs) with the size range from 30 to 150 nm. Exosomes carry various types of molecules originating from the cell-of-origin including proteins, nucleic acids, lipids, and metabolites. Exosomes also play essential roles in cellular communication and regulation (Reproduced from^[20]^).

Exosomes were first discovered by the Stahl and Johnstone groups in 1983^[15,16]^. Exosomes are small EVs with a size from 30-150 nm and can be produced by a majority of living cells^[17,18]^. Exosomes are secreted by exocytosis of multivesicular bodies and released into the intercellular environment^[19]^. As Figure 2 shows, hallmarks of exosomes include the tetraspanins (CD9, CD81, and CD63), ALG-2-interacting protein X (ALIX), and tumor susceptibility gene 101 (TSG101) protein^[20]^. The tetraspanins can serve as surface markers for exosome immuno-affinity isolation, and ALIX and TSG101 are commonly intravesicle biomarkers of exosomes^[21,22]^. In addition, exosomes are involved in many cellular functions such as metabolism and receptor transportation^[20,23]^, horizontal transfer of mRNA and miRNA^[24]^, and as a vector for oncogenic transfer^[10]^. Studies focused on exosomes include isolation and purification^[25-28]^, surface and intra-vesicle protein marker analysis^[29-32]^, cargo mRNA and miRNA analysis^[6,33,34]^, secretion and uptake pathways^[35-37]^, surface and cargo modification^[38-41]^, drug delivery^[42-44]^, and disease diagnosis and management^[45-47]^.

### Apoptotic bodies

Apoptotic bodies are generated as a result of programmed cell death and are primarily produced by cells undergoing apoptosis. Apoptosis occurs during cell-damaging or aging with the purpose of homeostasis. Cells can also show characteristic morphologies, including cell blebbing and shrinkage, nuclear fragmentation, and condensation/fragmentation of genetic material. Apoptotic bodies that are 500-1,000 nm in size are released as a product of apoptotic cell disassembly^[8]^. Like other types of EVs, apoptotic bodies contain protein, RNA, DNA, and other cellular fragments^[48-51]^. However, the only marker to recognize apoptotic bodies is phosphatidylserine (PS)^[52]^.Apoptotic bodies coordinate many cellular membrane molecular patterns, including high-mobility group box 1, heat shock protein 90, and interleukin-33 to facilitate cell blebbing^[53]^. Also, the caspase-mediated activation of pannexin 1 (PANX1) signal pathway serves as a “find-me” signal for phagocytosis and further apoptotic cell removal^[53]^. The receptor locating [PANX1^[54]^, CX_3_C-chemokine ligand 1 (CX_3_CL1)]^[55]^, and uptaking [phosphatidylserine (PtdSer)^[56]^, calreticulin (CRT)^[57]^] signaling pathway are well studied, but the detailed pathway on how cells are divided into small apoptotic bodies remains unraveled^[48,49,53]^.

## EVS AND THEIR MOLECULAR CONTENT

EV cargo mainly consists of various types of proteins and RNAs. Commonly found proteins in EVs are cytoskeletal, cytosolic, plasma membrane, and proteins that show post-translational modifications^[58]^. In addition, the tetraspanins, such as CD9, CD63, CD81, and CD82, have been found to be present in exosomes^[58]^. These transmembrane proteins are usually found on the surface of small EVs and can be used as targets for both small EV isolation and detection. However, recent studies have found that the tetraspanins can also be expressed on the surface of large EVs, including MVs and apoptotic bodies^[59,60]^. EVs can be secreted by most living cells, particularly tumor cells because of the continuous release and transfer of oncogenic information^[17,18]^. With the feature of containing host cells’ hallmark proteins, tumor-related markers can be expressed on both EV surfaces and within the vesicle^[61-63]^. 

The EV membrane also contains different types of receptors or ligands to trigger intracellular signaling pathways via a simple interaction in order to initiate an uptake process to deliver the enclosed information into target cells. The well-studied receptors and ligands pairs for EV uptake include the C-type to P-selectin glycoprotein ligand-1^[35,64]^, Galectins to Glycans^[65,66]^, mucins to galectin-3^[66,67]^, and PANX1 to purinergic receptor^[53,55]^.

RNA is also an important biomarker for disease management because of the function RNAs play in genetic regulation. The RNA content of EVs has been studied using such techniques as next generation RNA sequencing and RT-qPCR^[68]^. Many different types of RNAs have been found in EVs, including mRNA, non-coding RNA, miRNA, and tRNA^[58]^. mRNA is a widely studied RNA type found in EVs. Although cellular mRNA has about 400-12,000 molecules, EV mRNA typically has < 700 molecules and can be fragmented sections of mRNA and not full length transcripts^[69,70]^. Publications have shown that some types of mRNAs are only found inside EVs, but not expressed in the parental cells^[24,71,72]^.

miRNAs are small non-coding RNAs of about 22 nt in length. miRNAs are best known as gene silencing agents of complementary mRNAs and serve to regulate gene expression^[73,74]^. Because miRNA is associated with gene expression regulation, upregulated mRNAs may not be translated into the expected protein due to miRNA interference^[75]^. miRNA has been found in body fluids with complementary RNA-binding proteins that prevent enzymatic degradation^[76-78]^. With the same purpose as carrying mRNA, EVs also serve as vectors to transport miRNA to recipient cells^[68]^. EV-related miRNAs have been studied for cancer, such as miR-21 and miR-210^[79-81]^, and post-radiotherapy-related miRNAs such as miR-130a-3pand miR-92a-3p^[34,82]^. Understanding the RNA composition of EVs has become a critical endeavor for disease management.

## DIAGNOSTIC POTENTIAL OF EVS

Due to the valuable cargo EVs can carry, they have been widely studied as potential biomarkers for different diseases^[83-85]^. However, processes such as anticoagulation and endotoxin tube contamination can affect EV concentration in blood, which complicates enumeration data^[86-88]^. One advantage of EVs as a biomarker over many soluble molecules in the blood like hormones and cytokines is the inherent protection of the EV cargo from degradation, thus keeping the cargo intact and functional. Hence, EVs can be released from any location and into the bloodstream making them easily accessible for liquid biopsies. Additionally, the literature has shown that EV quantity, phenotype, or cargo content can change during disease progression^[89-92]^. Because tumor cells constantly release EVs, tumor-related EVs in plasma are at higher concentrations compared to normals^[89,93]^. Therefore, understanding tumor-related EV molecular profiles can help provide a fingerprint for precision medicine. 

EVs have also been studied as biomarkers for many non-cancer diseases, including diseases of the central nervous system^[94]^, liver (liver damage in viral hepatitis, hepatocyte injury in alcoholic, drug-induced, and inflammatory liver diseases)^[95]^, kidney (intrinsic kidney disease)^[96]^, brain (stroke)^[97]^, lung (Asthma)^[98]^, arteries (atherosclerosis)^[99]^ and radiation injury^[34]^.

## CONVENTIONAL METHODS OF EV ISOLATION

In order to analyze EVs’ cargo, EVs of interest must be isolated in high purity and high yields from body fluids because non-diseased cells also generate EVs that can mask subtle molecular signatures of disease. With increasing studies conducted on EVs, many techniques have been developed to isolate EVs from liquid biopsies. Some of these isolation techniques select the entire EV sub-types irrespective of the cells of origin and others can be specific so as to isolate only the disease-related EVs. In the next few sections, different conventional isolation strategies will be discussed.

### Precipitation and spin columns

**Figure 3:**
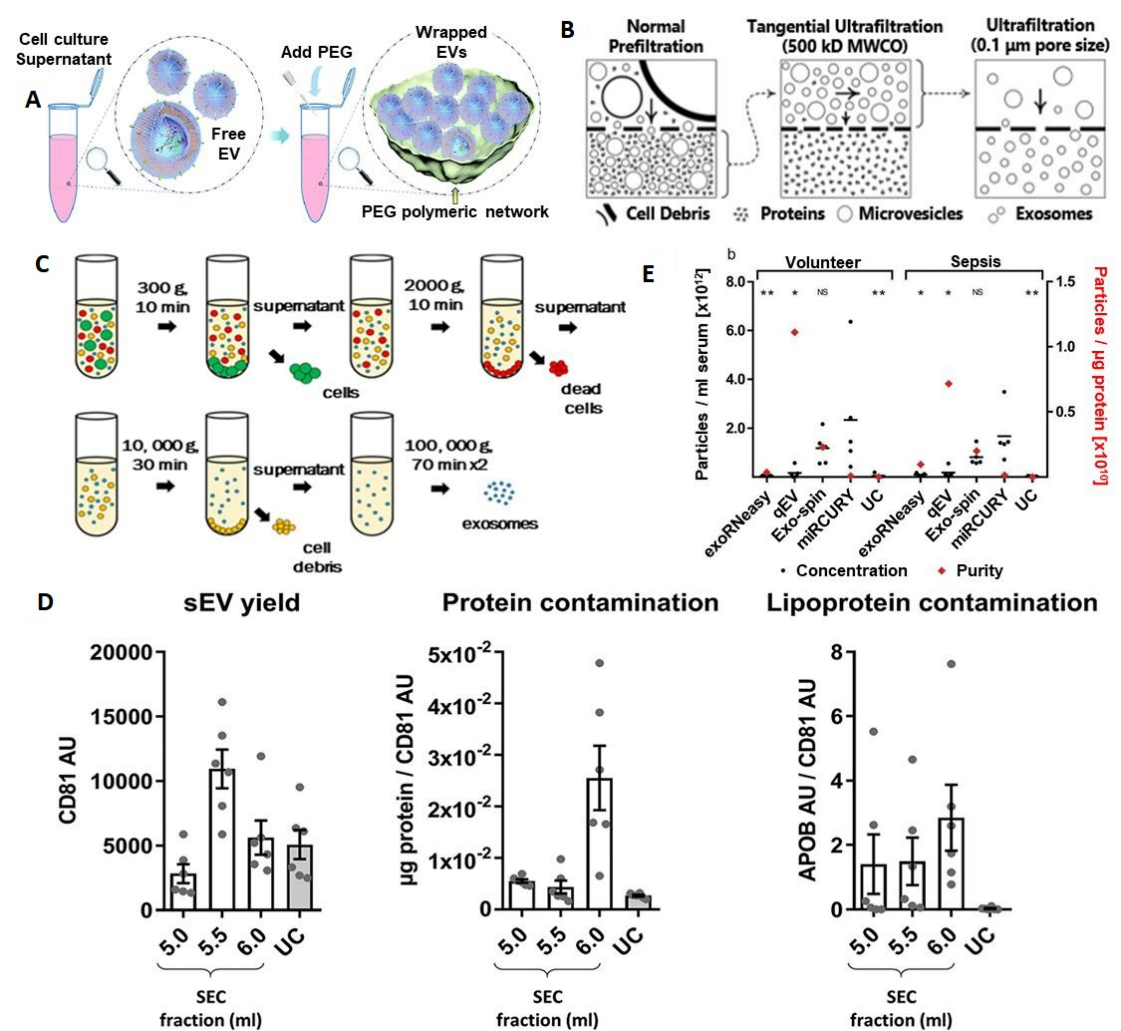
Conventional methods for EV enrichment. (A) Polymer-based enrichment: Precipitation with polyethylene glycol (PEG) (reproduced from Reference 100). (B) Filtration and ultrafiltration for EV isolation: normal prefiltration can collect sEVs and particles into the bottom layer of the culture dish. The bottom layer needs to be processed through tangential ultrafiltration, and the retentate is collected. Further ultrafiltration with expected pore size can be further processed and the EVs with a size smaller than the pore size will be present in the permeate (reproduced from^[28]^). (C) Ultracentrifugation for EV isolation (Reproduced from^[111]^). (D) Summary of yield and purity of sEVs isolated by SEC or UC: Normalization of APOB signal to CD81 content as an estimate of sEV purity from lipoproteins, also demonstrated almost 60 times higher APOB/CD81 ratio in the peak sEV fraction of SEC (5.5 ml) compared to the UC samples. SEC resulted in a higher yield of sEVs but with marked contamination by soluble protein and lipoproteins (reproduced from^[116]^). (E) Analysis of EVs by NTA demonstrates differences in size distribution. Black bars indicate the absolute number of vesicles isolated from 1 ml of serum; red diamonds plotted against the right x-axis represent vesicle purity defined as the particle to protein ratio. While precipitation most efficiently isolated EVs from serum, SEC-based isolation yielded fewer but more pure vesicles. Asterisks indicate significant differences in particle numbers compared to miRCURY. *P < 0.05; **P < 0.01; NS: not significant. All data are mean ±SD for five volunteers and five sepsis patients (reproduced from^[117]^). NTA: Nanoparticle tracking analysis.

Hydrophilic polymers, such as polyethylene glycol (PEG), reduce solubility by lowering the hydration of EVs and lead to precipitation^[100] ^[Figure 3A]. These kits can be used to separate EVs using lower spin speeds with higher yields compared to ultracentrifugation (UC). Upon addition of precipitation reagents, the solubility of proteins is also decreased^[101]^, and thus the isolate can contain protein impurities that can have a detrimental effect on downstream processing. Some of the advantages of precipitation reagents include preservation of EV integrity, no need for extensive equipment, selection pH close to the physiological range, and the possibility to process a large number of samples simultaneously^[102]^. However, poor reproducibility, impurities, and retention of polymer are a few drawbacks^[103-105]^. 

### Filtration

Filtration has been used as an isolation method for small particles based on size using nanomembranes^[28,106]^. Many times a sequential filtration or combined filtration with ultracentrifugation is used to provide high-grade exosomes [Figure 3B]. A modified polyethersulfone membrane is used for the pre-filtration of cell culture media, which can pass through the membrane. Then, tangential flow filtration with a 500 kD molecular weight cut off hollow fiber filter is used to filter out proteins. A final step with a low-pressure filtration can only make the desired size (smaller than pore size) of particles present in the retentate. Sequential filtration can generate a throughput of 0.96 mL/h, and the size distribution of isolated EVs can be controlled. However, clogging and shear stress can be applied to the particle, damaging the EV particle^[107,108]^.

### Ultracentrifugation

Ultracentrifugation is based on separation of particles according to their buoyant density. To affect the enrichment of EVs, several UC steps are typically undertaken. First, the particles with high buoyant density like cells (300-400g), cell debris (2000g), aggregates of biopolymers, apoptotic bodies, and other structures with a density higher than EVs are sedimented [Figure 3C]. The resulting supernatant with EVs is ultracentrifuged at > 100,000g for 2 h, which yields an EV pellet^[109-111]^.

In density gradient UC, a continuous density gradient including a sucrose or iodixanol density gradient and differential centrifugation is used^[111]^. In some cases, enriched EVs are further purified using filtration (0.1, 0.22 or 0.45 µm) or subsequent washing steps, which increases the purity of EVs but decreases the yield^[112,113]^. While UC can isolate EVs from large volumes of sample, some drawbacks include long isolation times (140-600 min), non-exosomal impurities, low reproducibility, and efficiency affected by the type of rotor, force, and sample type and only six samples can be processed in a cone ultracentrifuge^[109,113,114]^. Although UC methods yield low EV quantity compared to many other EV enrichment methods, Alvarez *et al.*^[115]^ has reported that UC with a sucrose density gradient yielded high purity. EVs of the size range 20-250 nm can be isolated by UC with the isolated EVs appropriate for assaying RNA and miRNA^[112]^. UC and size-exclusion chromatography (SEC) have been systematically compared for isolating small EVs (sEVs; exosomes) in rat plasma and results [Figure 3D] revealed that SEC-sEVs had higher particle number, protein content, particle/protein ratios and sEV marker signals than UC-sEVs. However, SEC-sEVs also contained greater amounts of APOB^+ ^lipoproteins and large quantities of non-sEV protein^[116]^.

### Comparison of different EV isolation techniques

Comparison of different EV isolation kits revealed that the total number of particles isolated from serum was the highest for miRCURY (precipitation), followed by Exo-spin (Size-exclusion chromatography), qEV (Size-exclusion chromatography), UC, and exoRNeasy [Figure 3E]. Also, SEC-based isolation yielded EVs with significantly higher particle-to-protein ratios than all other methods, indicating less co-isolation of soluble proteins. Isolates derived from precipitation and UC, on the other hand, displayed the lowest ratios due to increased protein contamination^[117]^. Side-by-side analysis of four kits also showed differences in performance. The size distribution of the isolated particles was appropriate (40-150 nm), and ExoQuick™ Exosome Precipitation Solution (EXQ) generated a relatively high yield of exosomes. However, albumin impurity was abundant for all the evaluated kits. There was significant correlation of the exosomal miRNA profile and specific miRNAs between kits, but with differences depending on methods. ExoRNeasy Serum/Plasma Midi Kit and EXQ performed better in the specific exosomal miRNAs recovery^[118]^.

### Affinity selection

EVs can contain protein makers that represent the cells from which the EVs originated. Tumor-derived EVs can express essential tumor-related proteins used for cancer disease diagnosis or progress monitoring^[58,119]^. By targeting specific proteins on the surface of EVs using immunoaffinity-based approaches, a specific type of EV can be collected. A variety of proteins can be targeted as biomarkers for EV isolation including the tetraspanins such as CD9, CD81, CD63, and cancer-related markers such as EpCAM, CD24, and CA125. Antibodies can be immobilized on a substrate such as the surface of a microplate or beads, and bind the EVs onto their surfaces only if they express an antigen specific to the capture antibody. Using immunoaffinity, the isolation can result in high specificity and purity for a particular EV subtype^[120,121]^. However, due to the cost of affinity-based assays, the isolation can only be applied with a small volume of sample, and EV-related proteins or RNA yields can be limited^[120,122]^.

The primary advantage of affinity isolation of EVs is that if the correct targeting surface antigen is used, the isolated EVs can be associated predominately to those that are disease-associated that can be advantageous for downstream molecular analysis. However, if the affinity isolation uses the tetraspanins, all EVs, in particular the exosomes and MVs, will be in the isolate.

## MICROFLUIDICS FOR EV ENRICHMENT

Many of the recently reported platforms for the isolation of EVs have been based on the use of microfluidics for several reasons including their ability to be integrated to post-enrichment processing steps such as enumeration and/or molecular profiling of the EV cargo. The enriched EVs can be enumerated^[123-128]^, surface and cargo proteins analyzed^[29,123,124,129-131]^, RNA profiled^[33,125,128]^, or diagnostics performed^[132-134]^. By including the appropriate micro-or nanoscale structures within the chip, approaches including affinity selection, filtration, centrifugation, viscoelasticity, and acoustic waves can be used for EV isolation using a microfluidic.

### Affinity enrichment

Affinity enrichment can enrich primarily disease-associated EVs, improving the quality of the molecular data secured from the isolate^[135]^. The ExoChip is an early example of a microfluidic used for affinity enriching EVs^[124]^. The ExoChip was fabricated using soft lithography and polydimethylsiloxane (PDMS) with surface-attached antibodies targeting CD63. Clinical serum samples were analyzed with immune-electron-microscopy and Western blotting used to confirm isolation of the EVs.

**Figure 4:**
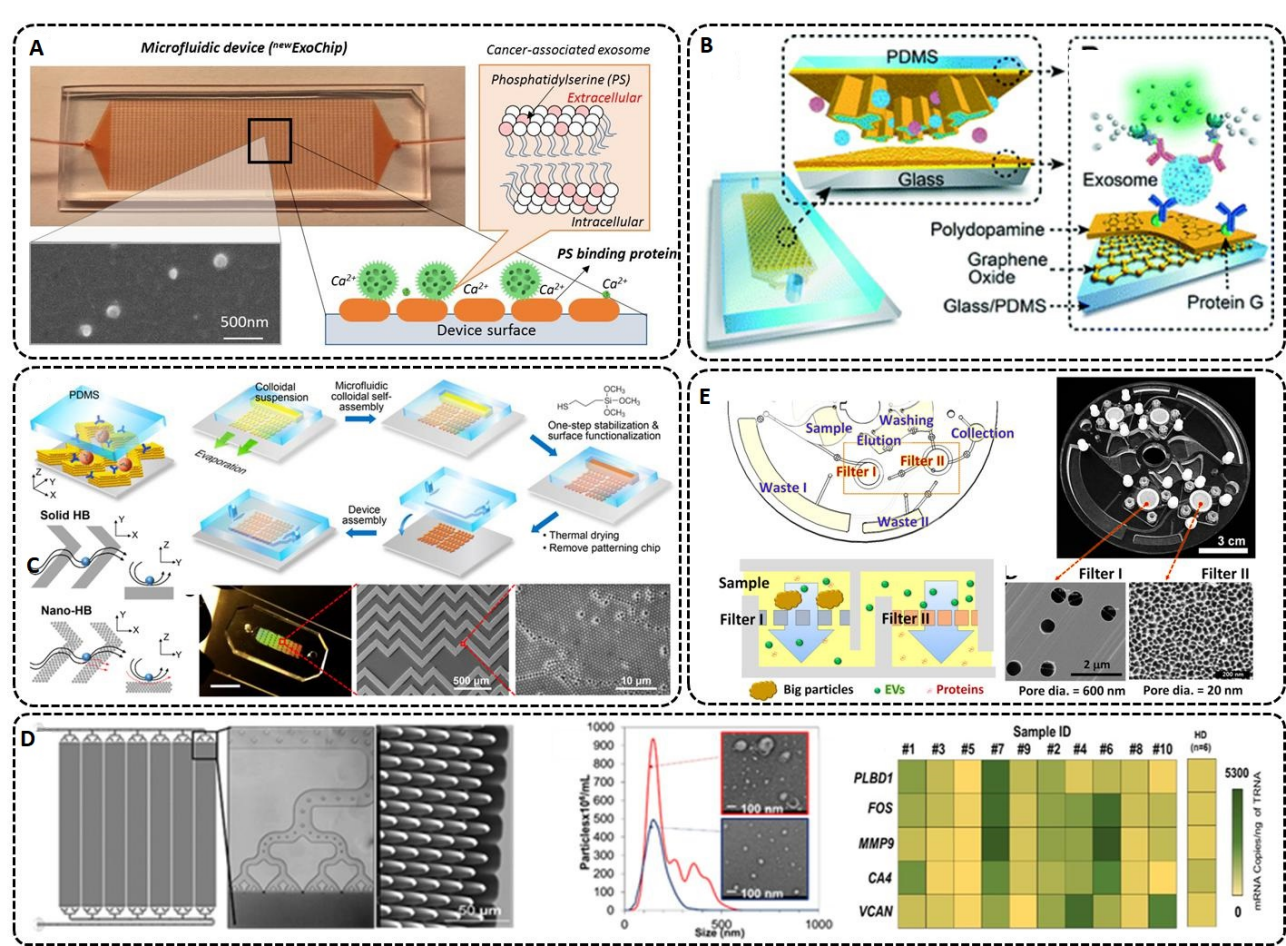
(A) ^new^ExoChip design, which features 30 × 60 circular patterns with a diameter of 500 μm in standard glass microscope slides. The mechanism of capture and release of cancer-associated exosomes using Ca^2+^-dependent binding between PS and annexin V and ethylenediaminetetraacetic acid (EDTA)-based Ca^2+^ chelation. The micrograph shows capture and released exosomes (reproduced from^[131]^). (B) Nano-interfaced microfluidic exosome platform (nano-IMEX). Schematic of a single-channel PDMS/glass device with expanded-view highlighting the coated PDMS chip containing an array of Y-shaped microposts. The surface of the channel and microposts coated with graphene oxide (GO) and polydopamine (PDA) as a nanostructured interface for the sandwich ELISA with fluorescence signal amplification (reproduced from^[123]^). (C) 3D herringbone nanopatterns are designed on a microfluidic device with the ability to detect tumor-associated EVs in plasma with a minimum of 200 vesicles per 20 µL. The nano-structures were used to increase the surface area, content mass transfer, and EV capturing speed, and reduce the hydrodynamic resistance (reproduced from^[129]^). (D) Microfluidic device made from cyclic olefin polymer (COP), which allows for high-rate production at a low cost to accommodate diagnostic applications. CAD drawing of a 7-bed EV Microfluidic Affinity Purification (EV-MAP) showing the distribution channels and the diamond-shaped micropillars of the device. NTA and TEM images of EVs isolated from a clinical sample by PEG precipitation and affinity selected with anti-CD8 mAb using the EV-MAP device. Heat map analysis of clinical samples (marked with numbers) and healthy donor for 5 genes whose up-regulation is associated with acute ischemic stroke (reproduced from^[139]^). (E) ExoDisc integrated system that combines a sequential filtration and centrifugation steps used for low viscosity fluids. The EVs are collected between filter I and filter II. The filters can be replaced with different pore sizes for different expected size range selection (reproduced from^[33]^).

Many microfluidic devices used EV-specific markers, such as the tetraspanins because in some cases disease-specific can be downregulated during disease progression. A newer version of the ExoChip (^new^ExoChip) used phosphatidylserine for enrichment^[131] ^[Figure 4A]. PS is expressed in the lipid bilayer of cancer-related EVs. The ^new^ExoChip achieved 90% capture efficiency of cancer-related EVs with the affinity-captured EVs released by Ca^2+^ chelation. 

A graphene oxide/polydopamine (GO/PDA) nano-interface was used to increase the EV capturing surface area^[123] ^[Figure 4B]. The capture antibody targeting CD81 and detection antibodies targeting CD81, CD63, and EpCAM were used to characterize the EVs and remove interferences from the sample. The assay provided a detection limit of 10^6^ particles/mL. Compared to the direct surface modification of GO or PDA only, the GO/PDA nano-matrix increased antibody capture efficiency of EVs by ~2-fold. 

An approach was reported using multiscale integration by designed self-assembly (MINDS) 3D nanostructures as the capture surface for EVs^[129] ^[Figure 4C]. With MINDS, flow streams can pass through a bumper structure and a nanostructured herringbone (nano-HB) results in enhanced contact time of the EVs with the capture surface. This offered a limit-of-detection of 10 EVs/µL and a total minimum detectable particle number of 200 per assay. For verification of the platform, 20 ovarian cancer patients and 10 non-cancer control plasma samples were processed, and differences were achieved between the two groups in terms of the number of enriched EVs.

It is difficult to mass-produce PDMS-based microfluidic devices^[136]^. As an alternative, thermoplastics are attractive because of their ability to be mass-produced and the simple modification protocols that can be employed to change their surface chemistry^[31,137,138]^. A cyclic olefin copolymer ^EV^HB-chip was manufactured with micro-injection molding and was designed to isolate tumor-specific EV-RNAs^[125]^. The herringbone structure was compared to a flat channel surface and the results indicated that the herringbone device captured ~60% more EVs. The device could process a wide range of sample volumes (100 µL to 5 mL) with a limit-of-detection of 100 EVs/µL. 

Another group developed a microfluidic device using thermoplastics made via micro-injection molding^[139]^. A 7-bed EV Microfluidic Affinity Purification (EV-MAP) device contained diamond-shape pillars [Figure 4D] with a 10 µm diameter and 10 µm spacing to allow for high throughput processing for enriching EVs via affinity selection (1.5 million pillars per chip). The device was used for diagnosing acute ischemic stroke patients using exosomal mRNA. mRNA expression of CD8+ EVs indicated that for genes upregulated during an ischemic stroke event, the EV-MAP device was successful in enriching EVs from clinical plasma samples, and gene profiling the EVs via droplet digital PCR for identifying stroke patients with a total processing assay time of 220 min. When the EVs were isolated using PEG precipitation, which isolates the entire EV subtypes, mRNA expression differences for stroke patients were not observed.

### Centrifugation and filtration enrichment

Filtration can be used as an EV microfluidic isolation method. An Exodisc was reported using a combination of centrifugal forces and nano-filtration^[33] ^[Figure 4E]. With a centrifugal force limit of 500g, EV sizes of 20-600 nm could be collected between two nano-filters. Filter I (600 nm pore size) was used to remove large particles, and Filter II (20 nm pore size) was used to enrich the EVs and exclude free proteins. The entire EV population was collected in 30 min with a recovery of 95%. Another platform with a combination of centrifugal force and filters was reported for inline EV detection by flow cytometry^[126]^. The EVs were isolated by anti-CD81 antibodies and with affinity microbead incubation, the enriched EVs could be concentrated and stained with a fluorescent dye. 

### Contactless EV enrichment methods

Researchers have also focused on contactless methods for EV enrichment using microfluidics, which takes advantage of the fluid associated with a microchannel and/or microstructures in the channel to affect the EV enrichment process. A microfluidic viscoelastic flow was developed for size-dependent isolation of EVs^[127]^. Poly(oxyethylene), PEO, was added into a sheath fluid at a concentration of 0.1% to maintain the feed solution at a particular viscosity. The particles were driven by an elastic force that situated particles in certain flow lines based on the size of the particle with larger particles traveling towards the center of the channel. The authors were able to demonstrate sEV recovery of ~80%. 

Microfluidic viscoelastic flow was also developed using an acousto-fluidic device for EV isolation^[128]^. The platform included two unique surface acoustic wave modules that were operated at 19.6 MHz for cell isolation and 39.4 MHz for EV isolation. The acoustic isolation was based on size because of the deflection caused by the acoustic pressure. The cell removal rate was > 99.999%, which resulted in 75% to 90% reduction of red blood cells. Using the modules in series, the isolation of 110 nm particles from whole blood yielded > 99% recovery, and the purity of the sEVs was ~98.4%.

## METHODS FOR EV DETECTION

Following isolation/enrichment of EVs, the EVs must be enumerated and their molecular content analyzed in many cases. For molecular cargo determinations, methods that can be used for protein or nucleic acid determinations include Western blotting, ELISA, RT-qPCR, and next generation sequencing. These methods rely on the disassembly of the EVs so as to analyze their intra-vesicular content. In spite of the high numbers of EVs found in clinical samples there are challenges when attempting to analyze their molecular content. For example, in spite of the exponential amplification of cDNA following reverse transcription, a certain mass of mRNA or miRNA must be secured to see a detectable signal. This is further complicated by the fact that most EVs do not contain full-length transcripts, and as such, the polyadenylated tail used for priming for the reverse transcription step may not be present and the yield of cDNA would be low. However, using random hexamer primers for reverse transcription as opposed to poly dT primers that bind to the polyadenylated tail of full length mRNA transcripts can address partially this challenge^[140,141]^. 

Because the molecular assay requires lysis of the EV to release the intra-vesicular content, the population and morphological properties of the EVs must be determined in advance of the molecular analyses. Therefore, it is necessary to conduct assays to assess population and morphological properties of the enriched EV fractions prior to the molecular assay.

A challenge with intact EV analysis includes the diverse size range of the vesicles (30-1000 nm), their low mass loads (for a 150 nm diameter vesicle, may contain ~10,000 nucleotides of various nucleic acids, and 10-100 protein molecules), and their relatively high particle numbers. As opposed to biological cells, which are 1-100 µm in diameter, special types of techniques must be used to characterize and count the intact vesicles due to their small size. For example, while conventional flow cytometry can be used for biological cells, variants of flow cytometry must be considered for enumerating EVs. In addition, while conventional Coulter counters can be used to enumerate biological cells, nano-Coulter counters must be used to enumerate EVs.

Current methods that can directly analyze EVs from a physical perspective include: (1) size and concentration analysis Nanoparticle tracking analysis (NTA), resistive pulse sensing (RPS), which can provide information on the size distribution of EVs and estimate concentrations; (2) surface protein expression analysis of EVs, which can determine the type and amount of protein expression by labeling with specific antibodies and fluorescent reporters that can permit the use of nano-flow cytometry; and (3) electron imaging of EVs. Direct imaging techniques include Transmission electron microscopy (TEM), Scanning electron microscopy (SEM), and atomic force microscopy (AFM), which can visualize the overall structure of the EVs including their size. In the sections that follow, a discussion on NTA, electron microscopies, nano-flow cytometry, and RPS will be provided for performing concentration and morphological analysis of EVs [Table 1].

**Table 1:** Comparison of EV detection techniques

**EV Detection Technique**	**Principle**	**Potential Advantage**	**Potential Disadvantage**
Nanoparticle Tracking Analysis (NTA)	Dynamic light scattering and Brownian motion	Straight forward operation; Both size variation and concentration information can be collected; Available addon parts for fully automatic operation	Sensitive to vibration; Contamination particles can also be included; High cost for the instrument and addon parts
Electron microscopies.	Electrons as the source of illumination	High-resolution images; Direct illumination for EV morphology	High cost for the instrument; Not appropriate for quantitative analysis; EV morphology may be damaged by the sample preparing steps
Atomic force microscopies	Scanning cantilever over the surface	High-resolution images; Ture 3D image with surface topology determinations	High cost for the instrument; EV morphology may be damaged by the scanning cantilever
High-Resolution Flow Cytometry	Light scattering or fluorescent excitation	Sub-type EV labeling and detection; Principle is applicable in micro/nano-fluidic technology for better sensitivity	Sensitivity limitation for particles size < 200 nm
Resistive pulse sensing (RPS)	Nanopore blockage with changes of current or potential	Higher sampling frequency comparing to optical sensing; Principle is applicable in micro/nano-fluidic technology for better sensitivity	Fabrication with intricate nano-structures, Small sampling efficiency; Calibration is required for each nanopore design

### Nanoparticle tracking analysis

**Figure 5:**
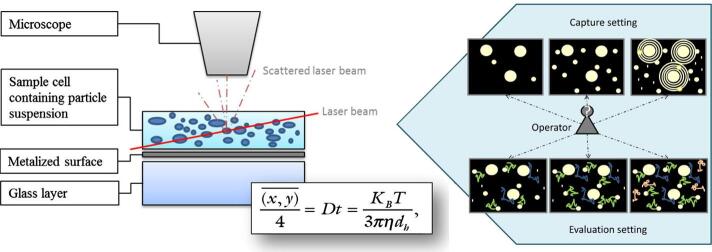
The principle of NTA measurements and the Stokes-Einstein equation for the analysis of particle size (reproduced from^[145]^).

NTA is a commonly used method for size and concentration determinations of EV samples^[142-144]^. Both dynamic light scattering and Brownian motion are the essential processes used to determine the size and concentration of particles using NTA. Figure 5 shows the measurement principles of NTA^[145]^. A laser beam illuminates the sample cell and the scattered laser beam travels through the objective of the microscope, which is analyzed by a CCD camera. The Brownian motion of each particle can be recorded and analyzed by the Stokes-Einstein equation [Figure 5], where D is the diffusion coefficient and calculated by the mean-square of particle movement, K_B_ is the Boltzmann’s constant, T is temperature, η is the solution viscosity, and d_h _is the particle diameter. From this equation, the particle’s d_h _can be calculated if the solution viscosity and temperature are known. In addition, by analyzing the particle presenting scattered radiation event frequency in each of the CCD image frames, concentration information can also be secured.

Considering the calculation is based on particle diffusion, NTA is typically useful for analyzing small particles with a size between 10 and 1,000 nm in diameter. NTA performance for monodispersed and polydispersed homogeneous particles has been confirmed in previously published work, while the performance for non-homogeneous particles, such as EVs or biological vesicles, is still under development^[146]^. 

**Figure 6:**
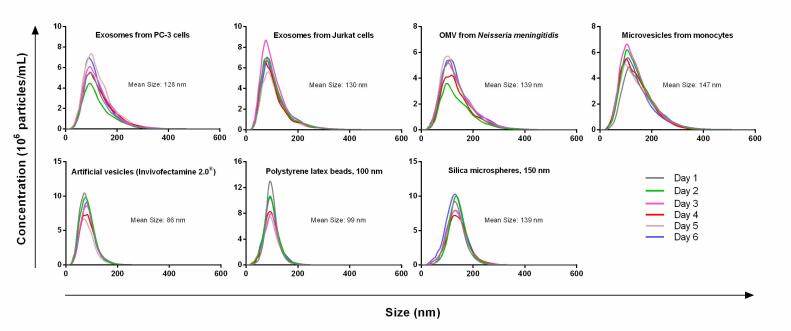
Concentration variations for different types of samples including EVs from PC-3 cell culture media, EVs from Jurkat cell culture media, Outer membrane vesicle from *Neisseria meningitidis*, microvesicles from monocytes, article vesicles, polystyrene latex beads (100 nm), and silica microspheres (150 nm). The samples were tested on 6 different days and the variation is from 1% to 18% (Reproduced from^[143]^).

In past studies, researchers have found that the introduction to a variety of parameters can increase the variability of results by up to 50%, including the threshold setting of the camera, the source of the EV sample, small vibrations, and even the method of operation^[143,144,147]^. Some state that sample dilution, camera grade, version of the analysis software, and the sample's size distribution should also be considered for an accurate EV size and concentration determination^[143,145,146]^. A study encompassing the detection and analysis of EV samples, microvesicle samples, artificial vesicle samples, polystyrene latex beads, and silica microspheres with NTA has been undertaken^[143] ^[Figure 6]. For artificial vesicles and polystyrene beads, the size variation and concentration were 3% and 9%, respectively. However, differences in the size of the EVs ranged from 1% to 6% and concentration varied from 5% to 18%. NTA also has some other drawbacks, such as a large sample size requirement (> 250 µL), an limited dynamic range (10^6^-10^9^ particles/mL), and only low viscosity samples can be analyzed, and need vibration free environment for analysis.

### Electron and Atomic force microscopies

**Figure 7:**
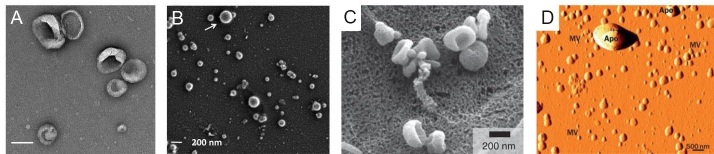
(A) Transmission electron microscopy image of EVs (scale bar = 100 nm). (B) Scanning electron microscope image of EVs showing the circular shape of the EVs (reproduced from^[151]^). (C) Scanning electron microscope image of EVs, which shows cup-shaped EVs (reproduced from^[150]^). (D) Atomic force microscope image for EVs (reproduced from^[156]^).

Electron microscopy can be used to image nanoscale samples, including EVs. In some cases, a perception bias may be introduced with imaging location selection, and it is also challenging to get an overall population estimation when the imaging areas are manually selected. However, electron microscopy, which includes TEM and SEM, is still a primary option when the morphology of EVs needs to be determined. Both electron microscopies use a beam of electrons, while TEM produces images using electrons transmitted through the sample and SEM analyzes the scattered electrons. TEM is most often used to collect information from the internal structure of the EV, while SEM can be used to interrogate surface structure. The resolution of both TEM and SEM can be as small as 1 nm^[148]^. However, the high-resolution advantage of TEM can be circumvented by sample preparation needs for EVs, which requires fixation and dehydration before imaging. Unlike cells with a cytoskeleton, EVs do not have an internal supporting structure. When the EV sample is dehydrated, the vesicle can form a cup-shape with loss of original morphology^[47,129,149,150] ^[Figure 7A and C]. Several studies have shown that EVs have a sphere-shaped morphology^[151] ^[Figure 7B]. Other papers have reported that EVs in SEM still show a cup-shaped morphology because the EV samples also undergo the same fixation and dehydration process [Figure 7C]. To overcome sample deformation, cryo-TEM can be used. For cryo -TEM, the sample can be placed in vitreous ice at the temperature of liquid nitrogen to eliminate the fixation and dehydration steps^[152,153]^.

AFM can record surface structure using a probe and laser reflection. A cantilever (i.e., probe) can deform while it interacts with the surface of the sample and the deformation of the probe caused by surface morphological changes can be sensed by laser reflection using position-sensitive photodiodes. AFM can obtain a true 3D image of surface structure and is commonly used for surface topology determinations^[154] ^[Figure 7D]. However, because EVs do not have an internal supporting structure, the vesicles tend to deform during sample preparation and imaging. For EV sample probe scans with monoclonal antibody immobilization are usually combined for better imaging quality^[155,156]^.

### High-resolution flow cytometry

**Figure 8:**
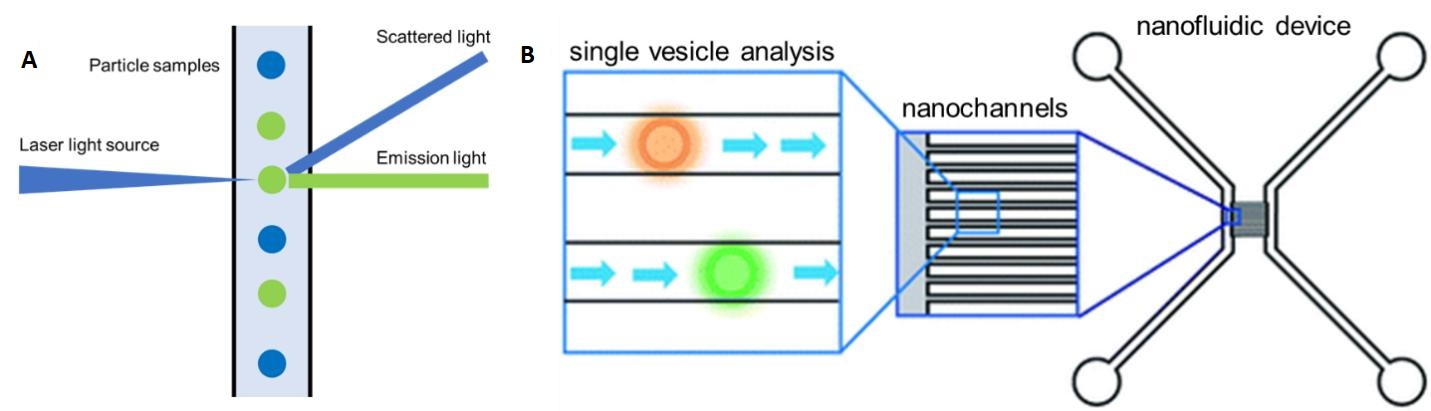
(A) The principle of flow cytometry. (B) A flow cytometry platform designed by Friedrich *et al*.^[164]^ The nanofluidic device contained 100 nanochannels with a width of 300 nm and the dye-labeled EVs could be sensed and recorded by a fluorescent microscope (reproduced from^[164]^).

Flow cytometry (FC) is frequently used for cell analysis providing the quantitative information of markers on the surface and internally to the cell. Conventional FC is typically used to analyze particles with a size > 300 nm. As Figure 8A shows, FC uses a laser beam with a specific wavelength, which impinges on a sample stream consisting of single particles arranged in a single file line generated by a sheath flow. The particles in the stream can scatter light from which critical information can be secured. For example, the scattered light can be used to determine particle size. Another functional mode of FC is fluorescence readout, which is typically produced by labeling certain cellular organelles or molecules with fluorescent labels. Because the specific biomarker is dye-labeled, FC can collect information that includes the expression level of the marker of interest. For example, FC can be used to analyze the cytotoxic T-lymphocyte related immune response by labeling CD8 expressing cells. 

In recent years, FC has also been applied for quantitative analysis of EVs. However, FC has a sensitivity limitation when it is applied to particles with a size smaller than 200 nm^[157-159]^. To overcome this drawback, the EV membrane is usually over labeled with a lipophilic dye, such as PKH26 or PKH74 to increase signal intensity^[160,161]^. The EV can also be analyzed indirectly when an adapter is applied^[162,163]^. The adapter typically carries a large quantity of fluorescent molecules to enable detection. Instead of directly sensing the EV, a well-calibrated adapter can provide higher intensity readouts by FC. 

Friedrich *et al*.^[164] ^developed a nanofluidic device to analyze EVs using FC [Figure 8B]. In this case, the sensing component consisted of a fluorescence microscope and an array of nanofluidic channels used as the flow cell. The device contained ~100 nanochannels with a size of 300 nm (width) × 300 nm (depth). Each nanochannel served as an individual FC sheath flow sampling unit and only 20 μL of sample was necessary for a typical measurement. The dynamic range of the nanofluidic device was from 10^10^ particles/mL to 10^14^ particles/mL. However, with this FC format, only concentration information of the EV sample was provided, but needed highly specific pre-isolation before sample readout^[159,165]^.

### Resistive pulse sensing

**Figure 9:**
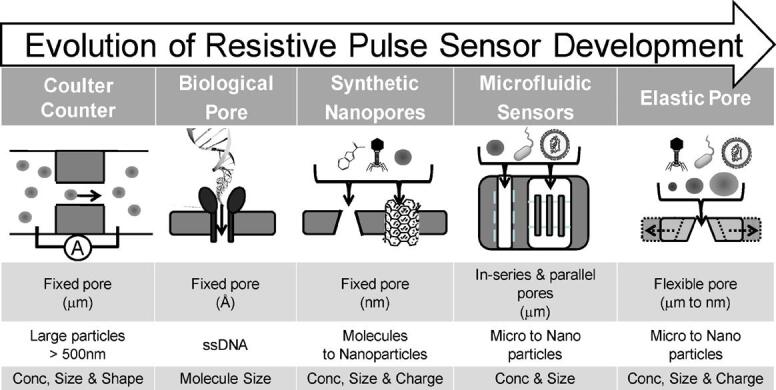
The evolution of resistive pulse sensing (RPS) from fixed pore with micro-scale to flexible pore with micro- to nano-scale sizes. RPS can also be applicable as a Coulter counter for EV analysis with the proper sized pore (reproduced from^[171]^).

RPS was first developed in 1976 for viral particle detection and characterization^[166,167]^. In 1996, Kasianowicz *et al*.^[168]^ utilized the biological nanopore as a Coulter counter for single-stranded DNA, and soon the RPS principle was applied to DNA sequencing due to different signal shapes of the four canonical DNA bases^[169,170]^. In recent years, RPS with flexible pore sizes and shapes has been used for EV concentration determinations and size analysis^[147,171] ^[Figure 9].

Equation 1.

**Figure:**
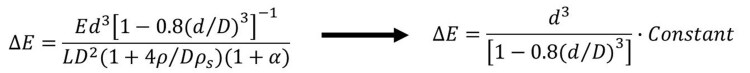


**Figure 10:**
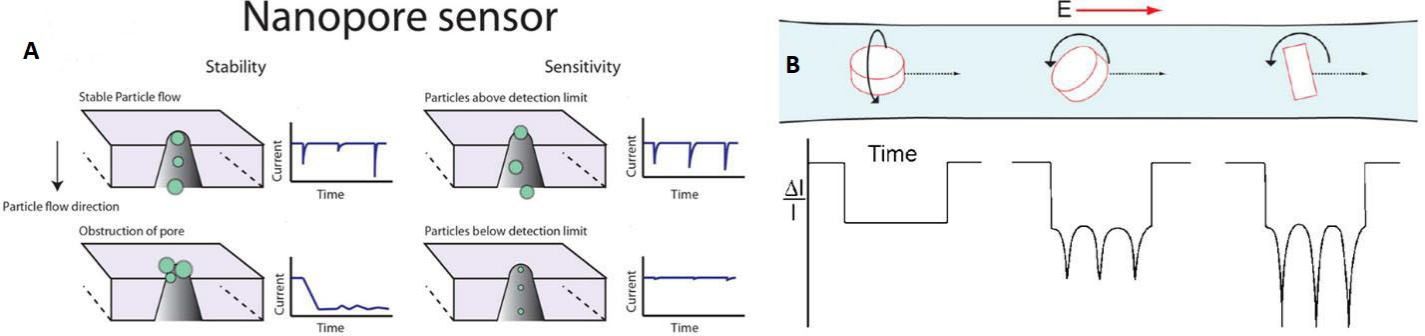
(A) The principle of RPS measurements with constant potential or current clamping across the measuring pore. The amplitude profile shows a relationship with particle size. The particle size distribution information can be determined by analyzing the event amplitude, and the event frequency can be analyzed for concentration information (reproduced from^[172]^). (B) The RPS is also used to study the particle shape, movement, and interaction with the solvent. The event can express the particle shape and also the rotation level and axis (Reproduced from^[183]^).

The RPS principles generate an output that can either be a change in potential or current measured across the nanopore structure. Whenever a particle moves through the nanopore, a proportion of the carrier electrolyte is replaced, which creates a change in the resistivity across the pore^[172] ^[Figure 10A]. The change in voltage across the pore can be described using Equation 1, where *ΔE* is the voltage change between the occupied and unoccupied pore, *E* is the applied potential, *ρ_s_* is the pore surface resistivity, α is the pore resistance to load resistance, *L* is the effective length of the nanopore, *d* is the particle diameter, *D* is the pore diameter, and *ρ* is the fluid resistivity^[166,167]^. For most cases, the majority of the parameters remain constant when a rigid pore and a homogeneous electrolyte are used for the RPS measurement. Thus, the size (*d*) of the particles in the sample can be determined by analyzing the amplitude of the electrical event (*ΔE*). In addition, with a known flow rate and event number, the concentration of the particles in the sample can be obtained as well. Equation 1 is primarily applicable to non-conductive particles because additional parameters must be considered for conductive particles including surface charge, particle charge density, and the permeability coefficient^[167,173]^. Also, for permeable biological vesicles, the particle resistivity may be lower than the carrier electrolyte due to the internal composition of the particle. As a result, some particles can produce the opposite polarity of signal compared to non-conductive particles^[173-175]^.

Synthetic RPS sensors can be fabricated in a controllable fashion, which generates the possibility of unique measurement opportunities compared to naturally occurring (i.e., biological) nanopores, such as altering the nanopore shape or placing nanopores in-series or in-parallel. The nanopore in-series can provide additional information about particle movement and generate the zeta potential of the particle. When the nanopores are placed in-series for monodispersed samples, the system can provide precise flow rate feedback, which can help to control the stream flow in real time^[176]^. When the nanopore in-series is used for polydisperse samples with a known flow rate, particles with different charge densities can provide different event duration. Nanopores in-parallel are another design strategy that can be used to increase sampling efficiency and throughput. When the nanopores are set up in parallel with individual electrodes, each nanopore will provide information simultaneously from the output circuit^[177-179]^. It is also feasible to couple the RPS with an EV isolation microfluidic chip that can be used to analyze the EV sample on-chip negating the need for off-loading the enriched EVs for analysis by NTA. With real-time electrical signal readout, RPS can provide EV sample information during the isolation/elution phases of the assay^[172,175]^.

Compared to optical sensing methods for EV quantification, such as NTA or flow cytometry, RPS can overcome some of their inherent drawbacks. For example, RPS can provide a faster sampling rate, up to 1000× higher^[143,172,180]^. For optical sensing, the exposure time has to be optimized to the millisecond or second timescale, which can make the sampling frequency ~1000 per second. The electrical signal recording for RPS can typically be set to 500 kHz^[181]^. In addition, the broad range of sampling frequencies for RPS can increase the dynamic range of the assay, from 10^5^ to 10^14^ particles/mL^[173,181,182]^. RPS is also used to collect information about particle shape and movement profile. Figure 10B shows a particle shape and movement signal trace demonstration that includes a disc-shaped particle translocation with different levels and axis of rotation^[173,183]^. 

RPS does have limitations for the detection of nanoscale particles. Firstly, because of the nanostructure the sampling efficiency and detection speed can be small with the majority of RPS platforms processing samples in the nanoliter to picolitre volume scale^[173,184]^. The nanopore in-parallel does make it possible to overcome this limitation, which can linearly increase the processed sample volume based on the number of pores in parallel^[173,185]^. On the other hand, increasing the through-pore transport speed or decreasing the sampling frequency and bandwidth can decrease the measurement sensitivity.

## CONCLUSIONS

As liquid biopsies are growing tremendously for applications in a variety of disease management scenarios, EVs are becoming an important target due to their biological and physical properties, including their relatively high abundance and the molecular information they carry^[186-189]^. EVs are rich in proteins and RNAs associated with their cell of origin, and these molecular markers have been shown to be useful for screening patients as well as being used to track disease progression^[30,190,191]^. As particles secreted by cells, EVs can easily pass through a series of biological barriers and travel throughout the circulatory system to their intended location^[18,134,192,193]^. This property allows EVs to be used as vehicles for drug delivery as well. EVs have also been studied as biomarkers for many non-cancer diseases, including the central nervous system^[94]^, liver (liver damage in-viral hepatitis, hepatocyte injury in alcoholic, drug-induced, and inflammatory liver diseases)^[95]^, kidney (intrinsic kidney disease)^[96]^, brain (stroke)^[97]^, lung (asthma)^[98]^, arteries (atherosclerosis)^[99]^, and radiation injury^[34]^. In any case, EV isolation/enrichment and quantification have become an important topic for both disease diagnostics and therapeutics. This arises from the fact that EVs are not just released from diseased cells, but non-diseased cells.

The challenge is that EVs must be enriched from a clinical sample prior to analysis of their molecular content and current methods for EV isolation are sometimes inefficient because they require a large volume of sample (UC) or alter the overall structure of the EV^[120,194-196]^. In addition, most traditional methods of EV isolation enrich the entire EV population consisting of both diseased and non-diseased EVs that can complicate the molecular analysis phase of the assay. Thus, the type of enrichment must be judicially chosen to match the application need. For example, if gene expression of the mRNA cargo from EVs is used for the application, endogenous expression of specific gene transcripts that may be found in non-diseased EVs must be taken into consideration because this may mask the gene expression from the mRNA found in diseased EVs only. Therefore, selection of EVs from the clinical sample using a highly specific disease-associated affinity agent may be required instead of using a non-specific enrichment protocol, such as UC. In addition, the clinical sample type and variation in sample collection and preservation may affect the quality of EVs selected and/or their yield during enrichment. As such, the EV field may need more detailed and clarified standardization protocols to minimize the variation between results emanating from different research laboratories^[197]^.

In many protein or nucleic acid assays using EVs for diagnostics (e.g., ELISA, PCR), high purity of the input sample is required to secure the necessary clinical information^[198-200]^. By applying micro/nanofluidic technology, high throughput and precise enrichment using affinity capture can secure a higher purity of disease associated EVs compared to many conventional or benchtop methods that isolate the entire EV population irrespective of cell-of-origin. However, these technology platforms are only capable of processing microliter sized sample inputs indicating that sampling statistics may be a concern or securing sufficient molecular cargo to feed into conventional molecular processing pipelines, such as NGS or even droplet digital PCR. Some microfluidic platforms that use label-free or contactless technologies can compensate for this shortfall of total process volume but at the expense of sample purity^[127,128,201]^.

In most cases, whatever molecular processing strategy is used, the intact EVs must be characterized including concentration, size distribution, and morphology, which are primarily based on electron microscopy, AFM, FC or NTA^[27,143]^. Recently, RPS has also been used for EV size and concentration determinations^[157,202]^. RPS, because it can be integrated into a microfluidic chip, can allow for straightforward analysis of particle physical characteristics and enumeration data following enrichment into a single device for potential point-of-care testing applications that use EV-based liquid biopsies.

## DECLARATIONS

### Copyright

**© **The Author(s) 2021.
