# Erratum: Isolation and analysis methods of extracellular vesicles (EVs)

**DOI:** 10.20517/evcna.2021.15

**Published:** 2021-09-15

**Authors:** Zheng Zhao, Harshani Wijerathne, Andrew K. Godwin, Steven A. Soper

**Affiliations:** ^1^Bioengineering Program, University of Kansas, Lawrence, KS 66045, USA.; ^2^Center of BioModular Multiscale Systems for Precision Medicine, Lawrence, KS 66045, USA.; ^3^Department of Mechanical Engineering, Temple University, Philadelphia, PA 19122, USA.; ^4^Department of Chemistry, University of Kansas, Lawrence, KS 66045, USA.; ^5^Department of Mechanical Engineering, University of Kansas, Lawrence, KS 66045, USA.; ^6^KU Cancer Center, University of Kansas Medical Center, Kansas City, KS 66160, USA.; ^7^Ulsan National Institute of Science & Technology, Ulju-gun, Ulsan 44919, South Korea.



The authors wish to make the following corrections to this paper^[1]^.

(1) In Figure 7A, a reference is missing, and the authors want to update it as follow:

**Figure 7:**
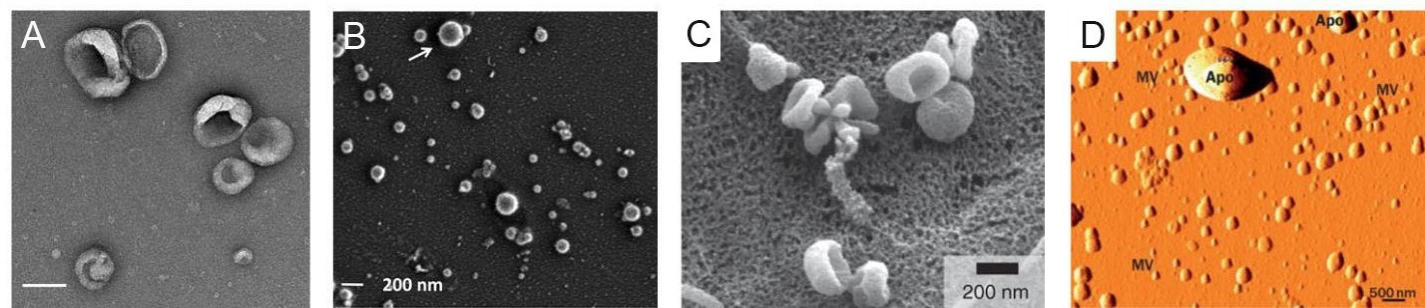
(A) Transmission electron microscopy image of EVs (scale bar = 100 nm)^[203]^. (B) Scanning electron microscope image of EVs showing the EVs’ circular shape (reproduced from^[151]^). (C) Scanning electron microscope image of EVs, which shows cup-shaped EVs (reproduced from^[150]^). (D) Atomic force microscope image for EVs (reproduced from^[156]^). (Figure 7A is produced from He *et al.*’s work with permission).

(2) The addition of a reference to the citation list:

203. He N, Thippabhotla S, Zhong C, et al. Nano Pom-poms prepared highly specific extracellular vesicles expand the detectable cancer biomarkers. *BioRxiv* 2021.

The authors apologize for any inconvenience caused and state that the scientific conclusions are unaffected. The original article has been updated.
